# Will the Real *Bemisia tabaci* Please Stand Up?

**DOI:** 10.1371/journal.pone.0050550

**Published:** 2012-11-28

**Authors:** Wee Tek Tay, Gregory A. Evans, Laura M. Boykin, Paul J. De Barro

**Affiliations:** 1 CSIRO Ecosystem Sciences, Canberra, Australian Capital Territory, Australia; 2 USDA APHIS NIS, BARC-West, Beltsville, Maryland, United States of America; 3 Bio-Protection Research Centre, Lincoln University, Lincoln, New Zealand; 4 CSIRO Ecosystem Sciences, Brisbane, Queensland, Australia; Biodiversity Insitute of Ontario - University of Guelph, Canada

## Abstract

Since Panayiotis Gennadius first identified the whitefly, *Aleyrodes tabaci* in 1889, there have been numerous revisions of the taxonomy of what has since become one of the world's most damaging insect pests. Most of the taxonomic revisions have been based on synonymising different species under the name *Bemisia tabaci*. It is now considered that there is sufficient biological, behavioural and molecular genetic data to support its being a cryptic species complex composed of at least 34 morphologically indistinguishable species. The first step in revising the taxonomy of this complex involves matching the *A. tabaci* collected in 1889 to one of the members of the species complex using molecular genetic data. To do this we extracted and then amplified a 496 bp fragment from the 3′ end of the mitochondrial DNA cytochrome oxidase one (mtCOI) gene belonging to a single whitefly taken from Gennadius' original 1889 collection. The sequence identity of this 123 year-old specimen enabled unambiguous assignment to a single haplotype known from 13 Mediterranean locations across Greece and Tunisia. This enabled us to unambiguously assign the Gennadius *A. tabaci* to the member of the *B. tabaci* cryptic species complex known as *Mediterranean* or as it is commonly, but erroneously referred to, as the ‘Q-biotype’. *Mediterranean* is therefore the real *B. tabaci*. This study demonstrates the importance of matching museum syntypes with known species to assist in the delimitation of cryptic species based on the organism's biology and molecular genetic data. This study is the first step towards the reclassification of *B. tabaci* which is central to an improved understanding how best to manage this globally important agricultural and horticultural insect pest complex.

## Introduction

In 1889, Panayiotis Gennadius, an Inspector of Agriculture, was sent to Agrinio, Greece to identify the small fly-like pest that was devastating tobacco crops there. After consulting with Filippo Silvestri and Adolfo Targioni Tozzetti, two of the leading experts on whiteflies at that time, it was determined that the pest was an undescribed species of whitefly and subsequently named, *Aleyrodes tabaci* Gennadius, later transferred to the genus *Bemisia* [1], [2]. For 104 years, the whitefly was considered to be a single, morphologically invariable cosmopolitan species, having an enormous host range and the ability to transmit a wide variety of plant viruses [3], [4]. From the late 1880s to the early 1980s, outbreaks were sporadic and relatively small, but this changed in the mid-1980s with widespread outbreaks occurring across the south western USA [5]. This was odd because this pest was well known across the region as a minor pest yet here it was destroying crops. Researchers concluded that while there were no morphological differences, there were sufficient molecular and biological differences to indicate that the outbreak pest was a different species [6]. This touched off a debate that has continued until today [7]–[10].

Species names are a tool for communicating our knowledge of species diversity and should make communication easier and clearer [11]. *Nomina dubia*, names of doubtful or unknown application as defined by the *International Code of Zoological Nomenclature* (ICZN, 1999), are problematic and hinder the communication of knowledge. These names arise when species are described based on a small number of specimens that are either entirely destroyed, uninformative or lost, and have original descriptions that are largely incomplete and do not provide characters needed for identification [12]. To avoid *nomina dubia*, the integrative taxonomy approach utilizes multiple lines of evidence to assist species delimitation and has been promoted as a solution [13]. The integrative taxonomic approach undertaken by the *B. tabaci* community has provided several lines of evidence indicating that a complete revision of the species complex is necessary. This evidence is summarized in [4] and draws upon a range of molecular, biological and behavioral data, all of which supports the conclusion that *B. tabaci* is a cryptic species complex. However, before initiating a taxonomic revision, extreme care needs to be taken so as to avoid further inaccurate species identifications [14], [15] and in the case of *B. tabaci*, this involves reconsidering the identity of the various species that have been synonymised under the name *B. tabaci* [16].

The debate over *B. tabaci* taxonomy began in January 1993 when Science [6] published a study that provided evidence suggesting that the whitefly causing considerable damage to food and fiber crops in the south western USA, while morphologically indistinguishable from *B. tabaci* (Gennadius), displayed considerable molecular and biological differences which together provided evidence that it was a distinct species and it was subsequently named *B. argentifolii* [6], [17]. In an attempt to draw a conclusion as to whether or not this was valid, Barinaga [7] posed the following question, “Is devastating whitefly invader really a new species?”, but by the article's end the search for a consensus remained elusive. A 1995 review of the subject again came to no conclusion [3].

Whether *B. tabaci* is a complex species or a species complex may seem a somewhat esoteric argument, but this is a major pest of some of the most important food and fiber crops. Ten percent of all known plant pathogenic viruses are transmitted by *B. tabaci* [18], [19]. The key staple crops cassava and sweetpotato are severely damaged by the viruses transmitted by this vector compounding the problem facing the achievement of food self-sufficiency across Africa, Asia and Central and South America [20]. Major cash crops in developing countries such as cotton, chilli and tomato are also devastated adding considerably to the financial burden borne by poor smallholder farmers in Africa, Asia and Central and South Americas as they borrow money to plant crops that are then subsequently wiped out [21], [22]. The answer as to whether we are dealing with a single vector species or complex of related, but different species will have a significant bearing on the applicability and transferability of management practices between regions where the pest occurs. These practices usually depend on insect biology, behavior, natural enemy interactions and responses to agricultural chemicals; what works for one may be ineffective for another [23].

The taxonomic identity of *B. tabaci* has a complicated history which has contributed to the confusion surrounding its identity. Whitefly taxonomy relies primarily on morphological characters of the fourth instar [24]. However, the morphology of the fourth instar in the case of *B. tabaci* was shown to be extremely plastic [24]. This meant that many of the morphological features used for identification of *B. tabaci* and 19 other *Bemisia* species that have been synonymized with it, have turned out to be highly plastic and a response to the host plant. This has no doubt contributed to the serial redescriptions and may well have been exacerbated by geographic isolation between the different taxonomists of the time, resulting in a lack of information exchange and the consequential assignment of multiple names to a globally distributed insect. As a result, between 1900 and 1978, 19 species of whiteflies were synonymised with *B. tabaci* [2], [16], [23], [25].

Since 1993, the body of knowledge surrounding this pest has grown to a point that in 2010 there was sufficient molecular data based on mitochondrial cytochrome oxidase I (mtCOI) to enable a compelling argument to be raised in support of the species complex argument [26]. This was later supported by a further study that applied a wider range of species delimitation metrics [11]. Using these metrics, it was proposed that rather than one species, *B. tabaci* was composed of at least 28 different morphologically indistinguishable species, all separated by at least 3.5% divergence in mtCOI [4]. To date 34 species have been delimited using these metrics [4], [27], [28]. In addition, delimitation is supported not only by available molecular evidence, but also all available mating compatibility studies. These have shown that crosses between individuals identified as different species using the delimitation metrics are reproductively isolated to the point that in most cases copulation does not occur and where it does, the resulting progeny are either sterile or reproductively inferior to their parents [29]–[45]. Given the debate is settling in favor of a species complex, thought now needs to be given to how best to revise the taxonomy of the complex. A key part of this revision will be to link named species that were synonymised under *B. tabaci* to the species identified using the delimitation metrics [14], [15]. The first step is to start at the beginning and determine the identity of the whitefly collected from tobacco in Agrinio, Greece, in 1889 [1]. This study shows how we succeeded in extracting DNA from an intact whitefly that was part of the 1889 collection and then amplifying the mtCOI and unambiguously matching it to a known member of the *B. tabaci* complex, and thus determining its true identity.

## Results

**Figure 1:**
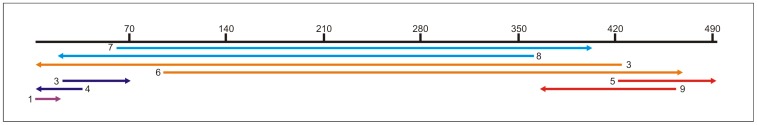
Schematic diagram detailing sequencing of the Gennadius 1889 *Bemisia tabaci* mtCOI amplicons using primers listed in Table 1. The final 496 bp contig was assembled from five amplicons (primer pairs 1+2; 3+4; 5+9; 3+6; 7+8).

**Figure 2:**
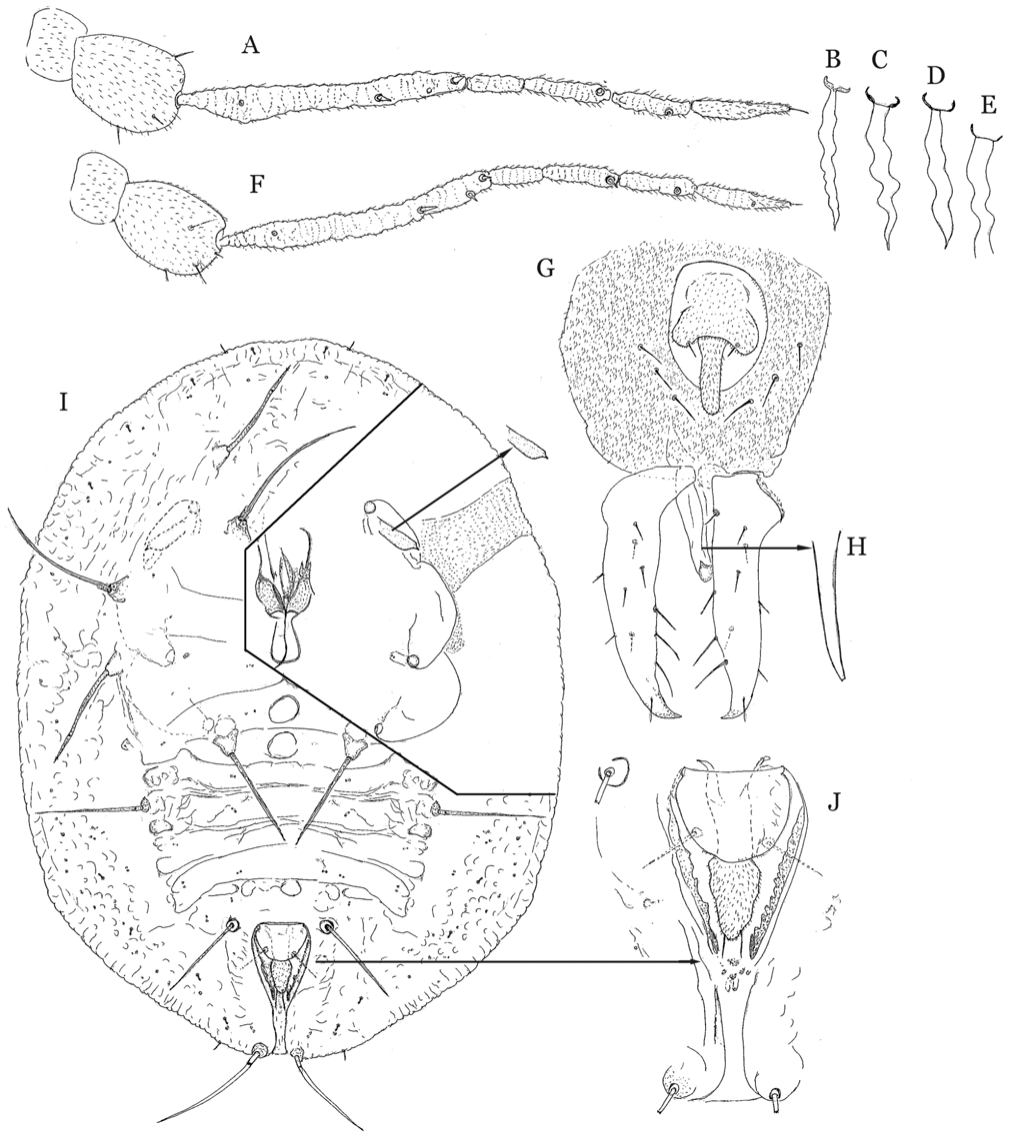
Illustrations of a puparium (I, J), variations in the cement gland of three adult females (C–E) and lateral view of aedeagus of an adult male (H) from *Bemisia tabaci* specimens collected by Gennadius from tobacco in Agrinio, Greece in 1889. In addition, illustrations of the antenna (A) and cement gland (B) of a female and the antenna (F) and posterior apex of an adult male (G) of specimens collected in China and determined to be *Mediterranean* based on DNA analysis.

We succeeded in amplifying five mtCOI fragments that assembled as a 469 bp of continuous sequence (GenBank JX268596) after primer site removal (Fig. 1). When aligned against the 34 consensus sequences representing the delimited species within the complex, the sequence was a 100% match with the consensus sequence for the *Mediterranean* species (Species names e.g. *Mediterranean*, *Middle East-Asia Minor 1* are currently being used until a formal process of renaming occurs and are those referred to in [26]). This species is commonly referred to as the Q biotype, although the biotype appellation is now largely discredited. The consensus sequence for *Mediterranean* was assembled from 87 different haplotypes all of which cluster within *Mediterranean*. Apart from *Mediterranean*, three other species in the complex occur in the Mediterranean region, *Middle East-Asia Minor 1*, *Italy*, and *Sub-Saharan Africa 2* and differ by 8.0%, 15.7% and 22.0%, respectively [26]. *Mediterranean* belongs to a cluster containing two close relatives in addition to *Middle East-Asia Minor 1*; *Middle East-Asia Minor 2* and *Indian Ocean* which differ by 5.7% and 9.8%, respectively (Fig. 2) [26]. The magnitude of these differences and the tight spread of within species variation (<3.5% divergence) enable unambiguously assignment of the Gennadius sequence to *Mediterranean*.

The sequence was then aligned against each of the haplotypes used to construct the *Mediterranean* consensus sequence. There was a 100% match with one haplotype only. When compared against those sequences in GenBank using a BLAST search, 13 GenBank records were recovered, HM807578 from Tunisia and DQ365857, DQ365858, DQ365860, DQ365862, DQ365863, DQ365865–DQ365871 all of which come from Greece [46]. One of these, DQ365865, came from a collection at Agrinio [47], 123 years after it was originally collected by Gennadius.

Illustrations are presented of a puparium (Fig. 2 I, J), variations in the cement gland of adult 3 adult females (Fig. 2 C, D, E), and lateral view of aedeagus of an adult male (H) from *Bemisia tabaci* specimens collected by Gennadius. In addition, illustrations were made of the antenna (Fig. 2 A) and cement gland (Fig. 2 B) of a female and the antenna (Fig. 2 F) and posterior apex of an adult male (Fig. 2 G) of specimens collected in China and determined to be *Mediterranean* based on DNA analysis. All of the puparia collected by Gennadius that were examined have 6 pairs of long, dorsal setae arising from tubercles, in addition to the long pair of A8 setae located lateral to the anterior margin of the vasiform orifice and a long pair of caudal setae (Fig. 2 I, J). However, the length of the dorsal setae has been shown to be extremely variable often due to the characteristics of the leaf on which the puparium is found. We hope to find morphological characters in the adult female and/or male, such as the shape and size of the cement gland, aedeagus and claspers, body setation and/or antennal characters that will be useful in distinguishing *Bemisia tabaci* and its look-alikes.

## Discussion

The differences in mtCOI between the different putative species make the assignment of the *B. tabaci* that Gennadius collected in 1889, unambiguous. We can therefore state without doubt that *Mediterranean* is the real *B. tabaci*. This species has now begun its own global journey of invasion spreading from its Mediterranean home range to at least 10 different countries [4], [48].

There have been several studies that have succeeded in amplifying mtCOI from museum specimens [49]. Only one has used members from the Hemiptera and these were samples that were less than four years old [50]. However, mtCOI has been amplified from beetles from museum collections dating back to 1820. In that study, of the 20 specimens assayed, five were as old as or older than our specimen and all yielded fragments [51]. So, our amplification of mtCOI from a member of the Hemiptera represents the oldest record.

To answer the original question posed by Barinaga [7] “Is the devastating whitefly invader really a new species?” the answer is yes, it is a new species. Further, Perring and co-workers [6] were right, they did have two different species. However, their assignment of the name *B. tabaci* to the indigenous American species (referred to as type A and later as the A biotype and now as *New World*) was incorrect as we now know that that this one belongs to the *New World* cluster of species [17], [26], [28]. But, in a way the ledger has now been squared. The true *B. tabaci* was not the one that invaded the USA in the early 1990s, that was *Middle East – Asia Minor 1* (MEAM1, formerly known as the B biotype or *B. argentifolii*), but rather the one first detected in the USA in December 2004 [48] more than 10 years after MEAM1 was detected and initiated the debate.

## Materials and Methods

### Sample Preparation

Specimens from Gennadius' syntype series are held as slide-mounted material in the Smithsonian National Museum of Natural History along with a collection of intact nymphs and adults on tobacco leaves, also collected from Agrinio, Greece in 1889 by Gennadius. The samples were provided free of charge by Gregory A. Evans (co-author) of the U.S. Department of Agriculture in Beltsville, MD.

We took the following precautions to assure that the sample being tested had no chance of contamination. The laboratory and the building housing the laboratory are stand alone and have never been involved with *B. tabaci* research. Furthermore, *B. tabaci* does not occur in the Australian Capital Territory where the CSIRO research facility undertaking the research is located. A single dried adult from the Gennadius 1889 collection was placed into 100% ethanol for 2 days. This adult had the unexpanded wings typical of a newly emerged adult and is most likely to have emerged from a nymph on the leaf. It was then transferred into a sterile 1.5 mL Eppendorf tube to air-dry for 10 minutes at room temperature. 50 µL of Invitrogen's UltraPure water was added to the insect and crushed using a pre-sealed sterile p200 tip and immediately used for PCR amplification.

### Primer design, DNA amplification and sequencing

PCR primers (Table 1) were designed using the mtCOI sequences derived from [26]. Primers were designed using the Oligo Primer Analysis Software v7.17 (Molecular Biology Insights, Inc., Cascade, CO 80809 USA) with the aim to minimise primer dimmers and hair-pin loops formation, reduced false priming sites, have an individual primer Tm of between 60°C to 72°C and to generate amplicons that ranged from 103 bp to 608 bp depending on primer pair combinations. All PCR reactions were carried out in a 25 µL reaction volume, and consisted of 2.5 µL of gDNA template, 1× ThermoPol Reaction Buffer (NEB), 0.625 u of DNA polymerase (NEB), 1.25 mM of dNTP's (Qiagen), and 0.4 mM of each forward and reverse primers [52]. A negative control that substituted the gDNA template with equal volume of UltraPure water was included in all PCR reaction preparations. PCR thermo-cycle profile consisted of an initial denaturing step at 95°C for 5 minutes, followed by 37cycles of template denaturing-primer annealing-template extension step of 95°C (30 seconds) - 54°C (30 seconds) – 72°C (45 seconds), and an additional template extension cycle at 72°C for 5 minutes. PCR amplicons were refrigerated at 10°C until the electrophoresis step, and at −20°C post electrophoresis. The presence of PCR amplicons were ascertained by running 6 µL of PCR reaction volume mixed with 1 µL of 6× Loading Dye (NEB) on 1.5% 1×TAE agarose gel at 90 V for 75 min. All agarose gels were pre-stained with GelRed (Biotium) following supplier's recommendation and viewed over a UV-transilluminator.

**Table 1:** Primer names, sequences and combinations used to obtain 496 bp of the mtCOI sequence from the Gennadius 1889 *Bemisia tabaci* specimen.

	Name	Primer sequence (5′→3′)	Primer Pairs	Amplicon (bp)
1	G1889-30F26	ATAATTTATGCTATANTRACTATYGG	1+2	103
2	G1889-110R23	AAGTGAARTAAGCTCGAGTATCT		
3	G1889-85F25	AYATATTTACWGTTGGRATAGATGT	3+4	103
4	G1889-163R25	GCAAGCCAACTAAAAATTTTAATTC		
5	G1889-466F24	TGTTYATTGGAGTAAAYTTAACTT	5+9	172
6	G1889-577R24	TACTYAAAATCCTRCCCGCARAAG	3+6	516
7	G1889-127F25	TCACTTCAGCTACTATGATTATTGC	7+8	388
8	G1889-493R22	AAACCAAGAAAATGCTGAGGAA		
9	G1889-606R32	TCTAAAACRATAAATAAAAAATAAATAACAGA		

Sanger sequencing of PCR amplicons was carried out by direct 1∶10 dilution with Ultra-Pure water, and using 2.5 µL of the diluted PCR amplicon in a final 10 µL sequencing reaction volume that consisted of 0.16 mM of appropriate PCR primer, 1 µL of BigDye reaction mix, and 0.75× BigDye sequencing Buffer (Applied Biosystems). Sequencing profile was 1 cycle at 96°C for 2 minutes, followed by 30 cycles of 96°C for 10 seconds, 50°C for 5 seconds, and 60°C for 4 minutes, and incubation at 4°C at the end of the PCR cycle. Post sequencing reaction clean-up was by adding 80 µL 75% room temperature isopropanol to each sample and spin at 18,400 rcf for 10 minutes, followed by washing with 250 µL of 75% room temperature ethanol and spin at 18,400 rcf for 5 minutes. Ethanol was discarded and samples dried on 55°C heating block for 10 minutes. Sequencing of all samples was by the ANU's ACRF Biomolecular Resource Facility. We used the Staden sequence assembly and analysis package to check for sequencing errors and construct our contig [53].

### Sequence identification

We used BioEdit to aligned against each of the consensus sequences that represent the 34 different putative species within the *B. tabaci* complex that have so far been identified [26]–[28]. The consensus sequences were designed from those haplotypes that were assigned to a particular putative species using Kimura 2 Parameter, Rosenberg's reciprocal monophyly (P(AB)), Rodrigo's (P(randomly distinct)), genealogical sorting index, and general mixed Yule- coalescent [11], [26]. The sequence was also compared against those in GenBank using BLAST.
